# Perphenazine in Treatment-Resistant Schizophrenia

**DOI:** 10.7759/cureus.51593

**Published:** 2024-01-03

**Authors:** Michael R Hower, Surya Kumar Karlapati, Anil K Bachu

**Affiliations:** 1 Psychiatry and Behavioral Sciences, Baptist Health - University of Arkansas for Medical Sciences, Little Rock, USA; 2 Psychiatry and Behavioral Sciences, Oregon State Hospital, Salem, USA; 3 Psychiatry and Behavioral Sciences, Allegheny Health Network, Pittsburgh, USA

**Keywords:** schizophrenia, antipsychotic management, paliperidone, aripiprazole, haloperidol, risperidone, inpatient psychiatry services, recurrent psychosis, psychosis, perphenazine

## Abstract

Antipsychotics are considered a gold standard treatment for schizophrenia. However, there is considerable variation in antipsychotic medication choice. Factors considered involved include symptomatology, prior response, and adverse reactions. This case report presents a 38-year-old male patient with schizophrenia in acute psychosis refractory to several antipsychotics. Hypotheses for the mechanism of action of antipsychotics and psychopharmacology are discussed, and treatment resistance is defined. The patient’s psychiatric, medical, and social history and past antipsychotic medications are reviewed. Afterward, the rationale for initiating perphenazine is discussed, and the patient’s improvement with this medication is examined. Current literature on perphenazine’s efficacy is also reviewed and discussed alongside its limitations.

## Introduction

Schizophrenia, as defined by the Diagnostic and Statistical Manual of Mental Disorders, V-Text Revision (DSM-5 TR), is a psychotic disorder characterized by the presence of two or more of the following symptoms, each present for a significant amount of time during one month, and at least one of symptoms (1-3): (1) delusions, (2) hallucinations, (3) disorganized speech, (4) grossly disorganized or catatonic behavior, and (5) negative symptoms [1]. These symptoms are associated with a significant impairment in quality of life, including work, self-care, or interpersonal relations. Continuous signs of these disturbances must be present for at least six months. These symptoms cannot be explained by substance use, medical conditions, or an underlying mood disorder, meaning depression or mania. 

Numerous hypotheses have been suggested for the cause of schizophrenia, including the “dopamine hypothesis,” which describes that specific receptors in the limbic system of the brain are dysregulated and hyperactive in schizophrenia, particularly dopamine 2 (D2) receptors, which are associated with movement, memory, learning, attention, and sleep [2]. These receptors are targeted “typical antipsychotics,” which include fluphenazine, haloperidol, perphenazine, and chlorpromazine. These typical antipsychotics can be further subdivided into potency based on affinity for D2 receptors, and their side effects include the potential to cause extrapyramidal symptoms (EPS), such as dystonia, akathisia, and long-term tardive dyskinesia [3].

With the advent of newer antipsychotic medicines, such as risperidone and olanzapine, that predominantly target the 5-HT_2A_ serotonin receptor shown to be non-inferior to typical antipsychotics, usage of many older typical antipsychotic medicines has fallen out of favor given the favorable side effect profile of atypical antipsychotics exhibits with regards to EPS [4]. However, the side effect profile of atypical antipsychotics includes metabolic side effects, including weight gain and elevations in blood sugar, potentially predisposing some patients to develop diabetes [3].

Treatment resistance occurs in an estimated 30% of individuals diagnosed with schizophrenia, and it is defined by The Journal of Clinical Psychiatry (2019) as minimum as a nonresponse trial of two antipsychotics of greater than six-week duration [5]. Nonresponse to antipsychotic therapy is defined as failure to improve psychotic symptoms and associated quality of life impairment. Given the significant variability in the literature, determining an adequate dosage for a trial is important. Therefore, we have added the additional criteria of the antipsychotic trials, equivalent to 600 mg of chlorpromazine, to improve validity as described in The Journal of Clinical Psychiatry [5]. Patients with treatment-resistant schizophrenia (TRS) have been found to have poorer outcomes in comparison to other patients with severe mental illness in activities of daily living, have lower marriage rates, and have persistent cognitive symptoms alongside positive and negative symptoms worsening their social functioning [5,6]. TRS is estimated to cost the US medical system an amount of 34 billion dollars a year, which is three- to 11-fold higher than patients with schizophrenia who aren’t TRS [6].

Although clozapine is generally considered a gold standard to be initiated after the failure of two adequate antipsychotic trials, challenges of clozapine maintenance therapy include continued blood draws, which are done weekly for the first six months, slow titration to a therapeutic dosage, and consistent follow-up needed to continue this medicine [7]. Furthermore, the Clinical Antipsychotic Trials of Intervention and Effectiveness (CATIE) and later the Cost Utility of the Latest Antipsychotic Drugs in Schizophrenia Study (CUtLASS) have demonstrated the superiority of clozapine in terms of improvement before discontinuation [8,9]. Data on TRS patients’ response to a non-clozapine antipsychotic is limited; however, one study notes that only 9% of patients defined as TRS responded to a third non-clozapine antipsychotic [7].

Several important factors in antipsychotic choice include the predominant presence of positive or negative symptoms, as it has been suggested that typical antipsychotics may be better at treating positive symptoms of Schizophrenia [10]. Prior response and side effects, for example, in a patient who experienced hyperprolactinemia or galactorrhea on risperidone, aripiprazole, could be chosen for its lower risk of these symptoms given its partial agonism of dopamine [3]. Antipsychotics’ effect on various receptors and binding affinity, meaning the strength of interaction between substance and receptor, is also an important consideration. For example, in a patient prone to psychotic agitation, antipsychotics that are more sedating, as defined by strength to antagonism of histamine-1 receptors (H1), such as chlorpromazine or olanzapine, may prove beneficial [3].

Perphenazine is classified as a medium-potency typical antipsychotic based on dosing equivalence; however, it has the second strongest binding affinity/to D2 receptors of 0.14 Kf [nM] out of all antipsychotics except thiothixene, which has Kf [nM] of 0.12 [11]. The Kf value measures binding affinity, and the smaller the Kf value of the number, the stronger the binding affinity. Perphenazine is dosed in 4-8 mg three times a day (TID) in schizophrenic patients; however, it can be dosed more aggressively in hospitalized patients up to 64 mg/day. Dosing can be adjusted to twice a day (BID) for outpatients with a larger dosage at night for its sedative properties [3]. Data in the CATIE trial found perphenazine to be as effective as atypical antipsychotics with minimal weight gain or metabolic concerns [3,8]. It is also available in formulary with amitriptyline, indicated for patients with schizophrenia and psychotic depression.

## Case presentation

Patient background

We present the case of a 38-year-old male patient admitted to a private non-profit hospital with a 12-year history of schizophrenia and more than eight hospitalizations over this time frame. The patient was noted to have no other medical or psychiatric conditions, and this patient’s medical history was negative for traumatic brain injury, seizure disorder, or major surgery. The patient is also noted to have a familial history of schizophrenia in his father and his paternal grandfather. Before his first psychotic break, he completed high school and two years of college, worked a full-time job, and attended to his ADLs independently. However, he has been unemployed since then, and his insight and ability to care for himself have deteriorated, given repeated hospitalizations, worsening psychosis, and the need for increased family support, which included his mother, sister, and brother, who lived with him. The patient was not married or in a relationship and did not utilize alcohol or any substances before his first psychotic break, which was confirmed by collateral. 

During this time, the patient has been tried on risperidone, paliperidone, paliperidone palmitate, haloperidol, olanzapine, quetiapine, and ziprasidone. Although medication compliance was frequently limited by the patient’s insight and outpatient follow-up, the patient did, however, meet the criteria for treatment resistance given two adequate trials of paliperidone palmitate long-acting 234 mg every four weeks and a separate trial of haloperidol 30 mg daily for three months, each over prior admissions and outpatient follow-up [5]. As 600 mg of chlorpromazine is equivalent to 12 mg of haloperidol and paliperidone and 12 mg of paliperidone is equivalent to 234 mg of paliperidone palmitate long-acting, these constitute adequate trials of these medications [12].

Per the patient’s psychiatric record review, the patient demonstrated a significant reduction in paranoia and aggression at home when given paliperidone palmitate but continued to exhibit markedly delusional thought content and negative symptoms and was unable to afford further dosages of this medicine after three months. In the case of haloperidol 30 mg daily over three months, although the patient’s positive symptoms improved, he continued to demonstrate significant symptoms, including disorganization and thought blocking, impairing his ability to communicate his needs.

Case presentation

On presentation to this hospital, the patient exhibited severe psychotic symptoms, including Cotard’s syndrome, characterized by believing his organs were deteriorating. Additionally, he displayed pronounced paranoid ideation that led him to become aggressive toward family at home, thought blocking, disorganization, and speech latency, rendering him unable to respond to basic questions on admission. Before this admission, the patient was living at home with his mother and siblings, had been unemployed for 12 years, and was single. Factors involved in this patient’s psychotic relapse include no outpatient follow-up for approximately six months due to uncontrolled psychosis, medication non-compliance, and substance use, as the patient was smoking one-fourth of an ounce of marijuana a day and had been drinking a bottle of alcohol a day for the past two weeks. The patient denied cocaine, methamphetamine, nicotine, opioid use, or prescription medicine misuse. 

**Figure 1:**
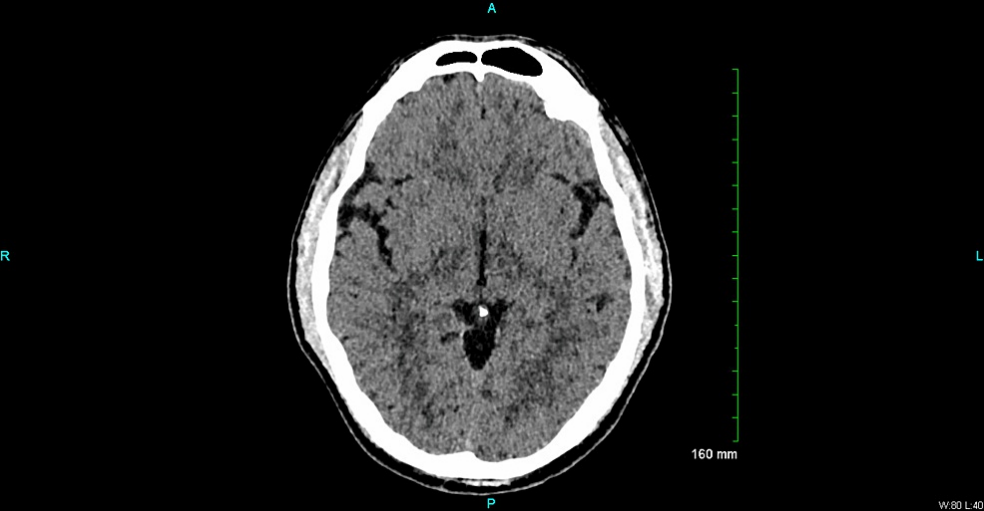
CT of the head

Clinical assessments, including a urine drug screen, were unremarkable, except for a moderately elevated blood alcohol level (BAL) of 0.152 and a mildly elevated aspartate aminotransferase (AST) level (Table 1). The patient’s Clinical Institute Withdrawal Assessment for Alcohol (CIWA) score was 0 on admission and throughout this patient’s hospital stay. CT of the head was ordered to evaluate for possible confounding Wernicke’s encephalopathy and negative for acute findings and changes in the mammillary bodies or periaqueductal gray matter consistent with Wernicke’s syndrome (Figure 1). No ophthalmoplegia or gait ataxia were noted on the physical exam. The medical team addressed potential alcohol withdrawal with thiamine and clinical monitoring, but lorazepam was not required. A thorough physical examination ruled out Wernicke or Korsakoff syndrome, and a review of childhood records showed no evidence of intellectual disability or cognitive delay. 

**Table 1:** Medical workup lab results

	Lab result	Normal range
WBC (K/cmm)	9.4	5.0-10.0
Hemoglobin (g/dL)	13.5	13.5-17.5
Mean corpuscular volume (fL)	89	80-100
Glucose POC (mg/dL)	82	75-110
AST (IU/L)	69	5-34
ALT (IU/L)	50	0-55
ALP (IU/L)	63	40-150
Bilirubin, total (mg/dL)	0.2	0.2-1.2
Bilirubin, direct (mg/dL)	0.1	0.0-0.5
BAL (g/dL)	0.132	N/A
Amphetamine (ng/mL)	73	0-500
Barbiturates (ng/mL)	0	0-200
Benzodiazepine (ng/mL)	0	0-200
Buprenorphine (ng/mL)	0	0-5
Cannabinoids (ng/mL)	0	0-50
Cocaine (ng/mL)	0	0-150
Opiates (ng/mL)	0	0-300
Oxycodone (ng/mL)	1	0-100
PCP (ng/mL)	0	0-25
Fentanyl (ng/ml)	0	0-1

Several medications were considered, given the patient’s complex presentation and inadequate response to previous treatments. Although clozapine is generally considered a gold standard to be initiated after the failure of two adequate antipsychotic trials, this patient also struggled significantly with outpatient follow-up and transportation, thereby making adherence to clozapine lab draws a limiting factor. Another option for antipsychotic choice would have been loxapine, given its profile similar to clozapine without limitations of weekly blood draws. However, given the patient’s marked positive symptoms, perphenazine was chosen instead. The severity of the patient’s symptoms was measured by the Positive and Negative Syndrome Scale (PANSS) of schizophrenia on the day of admission and two days before discharge (Table 2). The PANSS is a 30-itemized checklist where symptoms, including disorganization, hostility, and thought process, are graded from absent (+1) to extreme (+7) that is predictive of the severity of schizophrenia with scores of mildly ill, moderately, markedly, and severely ill-being equivalent to PANSS scores of 58-74, 75-94, 95-115, and 116 or more, respectively [13]. Before perphenazine’s initiation, the patient’s PANSS score was 184. 

**Table 2:** PANSS score on day of admission and two days prior to discharge PANSS, Positive and Negative Syndrome Scale

	Day of admission	Two days prior to discharge
Total PANSS score	184	77
Delusions	Extreme (+7)	Moderate (+4)
Conceptual disorganization	Extreme (+7)	Minimal (+2)
Excitement	Severe (+6)	Minimal (+2)
Grandiosity	Absent (+1)	Absent (+1)
Suspiciousness	Extreme (+7)	Minimal (+2)
Hostility	Extreme (+7)	Absent (+1)
Blunted affect	Extreme (+7)	Minimal (+2)
Emotional withdrawal	Extreme (+7)	Mild (+3)
Poor rapport	Extreme (+7)	Mild (+3)
Passive-apathetic social withdrawal	Extreme (+7)	Minimal (+2)
Difficulty in abstract thinking	Extreme (+7)	Moderate (+4)
lack of spontaneity and flow of conversation	Severe (+6)	Moderate-severe (+5)
Stereotyped thinking	Extreme (+7)	Moderate (+4)
Somatic concern	Extreme (+7)	Absent (+1)
Anxiety	Severe (+6)	Moderate (+4)
Guilt feelings	Extreme (+7)	Absent (+1)
Tension	Severe (+6)	Mild (+3)
Mannerisms and posturing	Severe (+6)	Mild (+3)
Depression	Moderate (+4)	Minimal (+2)
Motor retardation	Severe (+6)	Minimal (+2)
Uncooperativeness	Severe (+6)	Absent (+1)
Unusual thought content	Extreme (+7)	Mild (+3)
Disorientation	Extreme (+7)	Moderate-severe (+5)
Poor rapport	Extreme (+7)	Mild (+3)
Lack of judgment and insight	Extreme (+7)	Mild (+3)
Disturbance of volition	Moderate (+4)	Mild (+3)
Poor impulse control	Extreme (+7)	Mild (+3)
Preoccupation	Extreme (+7)	Minimal (+2)
Active social avoidance	Extreme (+7)	Mild (+3)

Perphenazine was initiated at 2 mg three times daily, with gradual titration to 4 mg three times daily over three days. Remarkably, this medication choice and adjustment significantly improved the patient’s thought process, allowing for meaningful conversations. Subsequent escalation to 12 mg twice daily over the next five days resulted in the complete resolution of Cotard’s syndrome and paranoid ideation. The patient’s repeat PANSS score two days before discharge was 77, a 41% reduction from baseline. However, in preparation for discharge, given the regional pharmacy shortage of perphenazine in dosages greater than 1-2 mg and the patient’s prior partial response to paliperidone, risperidone 0.5 mg twice daily was introduced to address any residual psychotic symptoms with no reported adverse effects or PANSS score changes.

## Discussion

Studies on perphenazine have been limited over the past 50 years despite its cost-effectiveness and availability, although current literature notes having similar tolerability to other antipsychotic medications [14]. Several studies have shown promise, including patients with perphenazine showing a lower rate of one-year readmission compared to haloperidol and risperidone [14]. It has also been perphenazine, and older antipsychotics may be as efficacious as SGAs for TRS and aripiprazole [14-16]. Compared to patients on low-potency antipsychotics, patients on perphenazine showed a 58% treatment response rate, while the low-potency group showed a response rate of 59% (RR, 0.97; CI, 0.74-1.26) [17]. When compared head-to-head with aripiprazole, 25% of perphenazine patients and 27% of aripiprazole patients showed clinically significant improvements as measured by PANSS score decreases of greater than or equal to 30% of baseline score [16]. Another study from 1965 showed a statistically significant difference favoring perphenazine over fluphenazine based on a reduction in psychotic symptoms [18]. A 2006 article in the American Journal of Psychiatry showed that perphenazine may be a more cost-effective alternative to atypical antipsychotics as measured head-to-head with haloperidol and risperidone based on readmission rates during one-year follow-up of treatment plus the overall cost of medication [19]. Out of the 202 evaluable patients, 26% of patients on perphenazine were readmitted, while readmission rates of patients on haloperidol and risperidone were 35% and 41%, respectively. The total estimated costs of risperidone were $20,317 yearly, nearly double that of perphenazine and haloperidol patients. With regards to perphenazine and its formulary with amitriptyline, one study noted higher global response rates to depression compared to amoxapine [20].

## Conclusions

Perphenazine is a cost-effective, well-tolerated alternative to mainstay antipsychotic medications with minimal metabolic side effects. Our patient presented with severe psychosis demonstrated a very favorable clinical response and was consistent with outpatient follow-up for two months after discharge. Long-term follow-up and trials of the long-acting version would be necessary next steps to explore, and this patient may have benefited from other antipsychotics, such as loxapine. Other limitations in this study include a lack of consensus regarding TRS and the need for further studies concerning antipsychotic management, augmentation, and combination therapy in TRS.
